# Probabilistic Models for Continuous Ontogenetic Transition Processes

**DOI:** 10.1371/journal.pone.0003677

**Published:** 2008-11-10

**Authors:** Anna Kuparinen, Robert B. O'Hara, Juha Merilä

**Affiliations:** 1 Ecological Genetics Research Unit, Department of Biological and Environmental Sciences, University of Helsinki, Helsinki, Finland; 2 Department of Mathematics and Statistics, University of Helsinki, Helsinki, Finland; University of Sheffield, United Kingdom

## Abstract

**Background:**

Probabilistic reaction norms (PRNs) are an extension of the concept of reaction norms, developed to account for stochasticity in ontogenetic transitions. However, logistic regression based PRNs are restricted to discrete time intervals, whereas previously proposed models for continuous transitions are demanding in terms of modelling effort and data needed.

**Methodology/Principal Findings:**

Here we introduce two alternative approaches for the probabilistic modelling of continuous ontogenetic transitions. The models are simplified in their description of forces underlying transitions, thus being empirical rather than mechanistic by their nature, but therefore applicable to situations where data and prior knowledge of transitions are limited. The models provide continuous time description of the transition pattern, insights into how it is affected by covariates, at the same time allowing for fine scale transition probability predictions. Performance of the models is demonstrated using empirical data on metamorphosis in common frogs (*Rana temporaria*) reared in a common garden experiment.

**Conclusions/Significance:**

As being user-friendly and methodologically easily accessible, the models introduced in this study aid the concept of probabilistic reaction norms becoming as general and applicable tool in the studies of life-history variation as the deterministic reaction norms are today.

## Introduction

Reaction norms are a common tool for describing phenotypic plasticity in quantitative traits [1]–[2] as well as for understanding evolutionary processes at the level of life-histories [3]. For ontogenetic life-history transitions, such as metamorphosis or maturation, reaction norms have traditionally been formulated deterministically, so as that an individual's developmental status is assumed to change exactly at the time the individual hits the reaction norm [4]–[5]. Although this kind of simplified formulation is undoubtedly useful when assessing how average phenotypes change in different environments, it lacks of realism in the respect that no stochasticity is assumed to be involved in the occurrence of transitions. This feature limits the utility of traditional reaction norms in applications incorporating demographic stochasticity and heterogeneity in the dynamics of life-histories and populations. These applications require realistic predictions for life-history events such as ontogenies [6].

To overcome this shortcoming, the concept of the probabilistic reaction norm (PRN) was developed by Heino et al. [7]. The idea of PRNs is that they characterize an ontogenetic transition process through transition probabilities, thus allowing randomness in the timing of individual transition events [7]. The estimation of PRNs was originally done based on the sizes-at-age of individuals, by assuming that this trait reflects environmental variation in the life-history transition process of interest [7]–[8] but then later on expanded to encompass information on any relevant covariate [9]–[11]. In practice the estimation of the PRN is done through a logistic regression, so that probabilities for ontogenetic life-history transitions are estimated separately for discrete time intervals [7]:
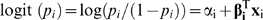
(1)where *i* is an index of time-interval, *p_i_* is the transition probability, α_i_ is an intercept, **β_i_** is a vector of free model parameters, and **x**_i_ is a vector of explanatory variables. The time-intervals may be determined either by natural periodicity in data (e.g. annual reproduction season), or be set artificially by investigators by grouping continuous time observations into discrete time intervals [7]. In latter case intervals must be set wide enough to contain sufficient data to reflect the underlying transition pattern. Therefore, the logistic regression based PRNs model is not able to predict ontogenetic transitions in continuous time or within fine scale time intervals. While this has not been viewed as a problem in the analyses to which PRNs (the model developed by Heino et al. [7] or its demographic analogy developed by Barot et al. [8]) have almost exclusively been applied to, i.e. age and size at maturation of fish stocks with an annual reproductive cycle (e.g. [12]–[13]), in the case of more rapid developmental processes, such as metamorphoses, obvious limitations arise.

These problems were first addressed by van Dooren et al. [14], who introduced a survival analyses and path-integration based approach for making a connection between age and size dependent continuous time maturation rates and discrete time PRNs for the age and size at maturation. However, any practical implementation of this method has proven challenging as full ontogenetic trajectories should be known (or assumed), but data for this hardly ever is available from natural populations [9].

Here, we introduce two alternative survival analyses based modelling approaches for describing ontogenetic transition processes in continuous time. These models are simplified as compared to the approach developed by van Dooren et al. [14], but at the same time less demanding in terms of modelling effort and data requirements. Therefore, they provide user-friendly and broadly applicable tools for continuous time analyses for typical ontogenic transition data sets, as well as for predicting transitions at very fine time scales. Performance of the models is demonstrated using empirical data on timing of metamorphosis in the common frog (*Rana temporaria*).

## Analysis

### Survival analysis based approaches for ontogenetic life-history transitions

Ontogenetic transition processes are of a type where an individual ages, and at some point in time experiences an event that can only occur once for each individual. In medicine, this kind of process is investigated using *survival analyses*, the name deriving from the fact that the considered event is often death (e.g. [15]). The starting point for survival analysis is to assume that the probability that an event will occur is governed by a rate *h(t)*, which is usually called the *hazard*. If the mathematical form for how this changes with *t* is known, then the probability that the event (e.g. maturation or death) will not occur before time *t*, denoted as *S*(*t*) and called the *survivor function*, can be calculated by
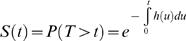
(2)Or, if time is discrete, the probability that nothing happens before time *t* is the product of the probabilities of nothing happening in each time step before *t*. Typically, survival analyses use data on the times to events (*T*) to ask how the hazard function or, equivalently, the survivor function is affected by different covariates.

In their continuous time description for maturation process van Dooren et al. [14] focused on the analytical form of the hazard function, specifically, how the hazard changes with age (i.e. time) and size of an individual. In this approach, the age-at-size trajectory of the individual either have to be known exactly or the size can be expressed as an analytical function of age. More generally, the hazard function can depend on any relevant covariate and, if it changes in time, then the time-path or the value of the covariate as an analytical function of time must be incorporated to the model. However, a typical case might be that most what is known about an ontogenetic transitions process is the observed pattern of transitions themselves, but little information is available about the underlying covariates, especially how their impact changes in time and functions to describe their effects on the transitions. In such a case formulating the hazard as a function of all the covariates and further finding analytical formulations for their time-dependencies can be very challenging and time-consuming.

In the following, we will propose two alternative modelling approaches that will relax the effort of composing the exact analytical form of the hazard, by directly making assumptions about the survivor function, and how it is affected by the covariates. The models are qualitatively very similar, but depending on the particular research question and the data at hand, one may be more convenient that the other.

#### 1) Parametric Survival Analysis

As shown above, if the hazard function can be assumed to take a particular mathematical form, then the survivor function can be calculated (eqn 2). In the approach formulated by van Dooren et al. [14] the hazard function was described as depending on the age and size of an individual, and the size was then described as a deterministic function of age. The hazard function then takes the form *h(t)* = *f_1_(age)*+*f_2_(size)* = *f_1_(age)*+*f_2_(g(age))*, where *f_1_* and *f_2_* can be any analytical functions and *g* is the deterministic growth function, so that in the end the hazard function is formulated being a function of time. Obviously the same also holds for any other time dependent covariate. In general, different distributions for survival times and corresponding analytical solutions for survivor functions are linked to particular time-dependent formulations of the hazard functions. Thus, instead of explicitly formulating the hazard function, as suggested by van Dooren et al. [14], one can simply approximate it with a formulation that directly yields a known distribution for survival times (a table of distributions and their underlying hazard functions is given e.g. in [15]). For example, for Weibull-distributed survival times the hazard is given by *h(t)* = γλ*t* ^γ−1^, which, depending of the choice of γ can be either an increasing, decreasing or constant function of time. The relationship between size (or any other time dependent covariate) and time may not be deterministic, but this is accounted for by the random part of the survival curve, yielding a distribution of survival times. Overall, by not using intermediate measures of time dependent covariates, this approach looses precision but gains in applicability (because it does not need intermediate measures).

The modelling effort reduces to a parametric survival regression in which the distribution of an individual's survival time *T* (i.e. the time it takes until an individual faces the transition event) is modelled directly by [15]–[16]

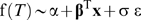
(3)where α is an intercept parameter, **β** is a vector of free model parameters, **x** is a vector of optional covariates, σ is a scale parameter (also a free model parameter) describing variance in the data, ε is a random variable, following some distribution, and f is a link function appropriate for the distribution of given ε. Several choices exist for the distribution of ε, with those giving a logistic, Weibull, Gaussian or exponential distribution for *T* being popular. In practice, this model estimates the shape of the distribution for *T* and then covariates shifts the location of this distribution along the time axis. The underlying assumption then is that covariates do not change the shape of the survivor function itself but only its location, in proportion to the relative effects of the covariates. Therefore, the estimated parameters of eqn (2) can be used directly for assessing how much variation is induced to the timing of the transition event by the covariates.

Once the parameters of the survivor function have been estimated, the model can be used to predict the occurrence of transitions. For example, for any two time points *T_1_*≤*T_2_* the probability of a transition event within the interval (*T_1_*, *T_2_*) is (P(*T*<*T_2_*)−P(*T*<*T_1_*))/(1−P(*T*<*T_1_*)), where the probabilities are derived from the distribution function of *T* estimated by the parametric survival model (eqn 2). Calculated for a time interval, the transition probability corresponds to the concept of traditional discrete time PRNs, but the fundamental difference between the two methods is that the PRN approach only provides predictions for pre-fixed, not too narrow intervals, whereas the continuous time survivor function provides estimates for any freely chosen time interval, allowing for fine-scale predictions.

#### 2) Semi-parametric Survival Analysis

One might not be directly interested in the survival function (distribution function of *T*) itself, but on how it is influenced by the covariates. Cox [17] developed an elegant method for the analysis of survival data by splitting the model into two parts: 1) the survival function, which only depends on time, and 2) a term for the ratio of the hazards (i.e. rates of the events) for different classes. He showed that under this model the relative rates of the hazards do not depend on the actual shape of the hazard function, but the covariates shift the hazard up or down by the same proportion. Hence, the model is called the *proportional hazards model*. This modelling approach is particularly convenient if interest lies on the proportional effects of the covariates on *T*, rather than the distribution of *T* itself. The Cox proportional hazards model assumes that the effects of the covariates are multiplicative, so that the survival probability S(*t*) = P(*T*>*t*) is given by
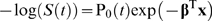
(4)where **β** and **x** are as above, *t* is any freely chosen time point, and P_0_ (*t*) is a baseline hazard function that gives the probability P(*T*≤*t* | **x** = 0). Defined in this manner, P_0_ may seem biologically meaningless, but this is not the case. In case of categorical covariates one of the categories can be set represent a baseline, which is then increased or decreased by the other categories. If a covariate is continuous, then it can be rescaled so that the variable value describes the deviation from a baseline value. For example, the baseline can be set to represent an average individual, or there may be some physiological thresholds that an individual has to reach before it can metamorphose or mature [18]–[19]. Cox regression is very convenient in the sense that no underlying distribution for the transition time needs to be assumed. Similarly, when assessing the proportional effects of the explanatory variables on S(*t*), P_0_ does not have to be known either. It is only required for estimating the actual survival probability S(*t*) = P(*T*>*t*). From these, predictions for transition probabilities can be derived for any time interval similarly as above.

The choice between the parametric (eqn 3) and the semi-parametric (eqn 4) survival models depends on the study question. The parametric model may often be preferable over the semi-parametric one as the former produces a probability distribution for *T*. However, interest may sometimes be focused on the amount of phenotypic variation induced by different variables, or there may be a biologically meaningful baseline for a developmental process that is then modified by the environment. In such cases the semi-parametric model may be preferred. Both the survival models developed above are easily accessible in most statistical packages, such as R [20] and SAS [21].

One common problem in survival analysis is that the exact timing of the event may not be known. In this case the data are described as *censored*. For example, if the event has not occurred before the end of a trial (i.e. the individual survives beyond the experiment), the datum is described as right censored. Of more relevance here is interval censoring. This is when the event is known to occur within an interval (e.g. between two sampling periods), but the exact time of the event is not known. For example, in the context of maturation, this would be seen in data where a large sample of individuals was drawn from a population, and the numbers of mature and immature individuals were counted. If the data is interval censored, parametric survival model (eqn 3) still produces continuous time model for the underlying transition process. In case of semi-parametric survival model (eqn 4) *T* would be replaced with an ordered factor indicating the time interval in which case the survivor function in eqn 4 is replaced with 1−ϕ(*t_i_*), the complement of the transition probability for a time interval *i* [22].

### Further model extensions

The exact interpretation of reaction norms is that they define individual phenotypes under specific environments [7]. However, PRNs and survivor functions are still estimated based on information from many individuals sampled from one population (eqn 1, 3 and 4; [7]–[8]). Still, it is likely that there is variation between individuals in their own reaction norms, which needs to be accounted for. This is because interest in reaction norms partly springs from the fact they would allow separation of genetic and environmental influences on life history transitions. To this end, direct estimation of genetic variation in ontogenetic transitions would be of interest [14] and for the survival models presented above tools (and also software solutions) for this are readily available [20], [23]–[24].

Individual random effects can be included into survival analysis, where they are termed *frailties* [15]. This is done using a hierarchical model, i.e. by assuming that the frailties represent random effects drawn from some distribution. Frailties other than individual effects can also be added, for example adding a sire effect can be used to estimate the amount of additive genetic variance in transition probabilities, which in turn can be used to predict the trait's response to selection [25]. Because the model (in particular equation 3) is linear, several random effects can be included into the same model in the same way, so for example a full quantitative genetic model can also be fitted (e.g. [26]).

### Model performance

To demonstrate the performance of the survival analysis based models for ontogenetic life-history transitions developed above (eqns 3 and 4), we focused on modelling the timing of metamorphoses of the common frog (*Rana temporaria* L.). This was based on the metamorphosis events observed in a laboratory experiment in which tadpoles were exposed to different, controlled environmental treatments. The benefit of using this kind of data is that the sources and magnitude of environmental variation in the timing of metamorphoses are known, and thus we were able assess whether the survival models were able to detect and predict them.

**Figure 1:**
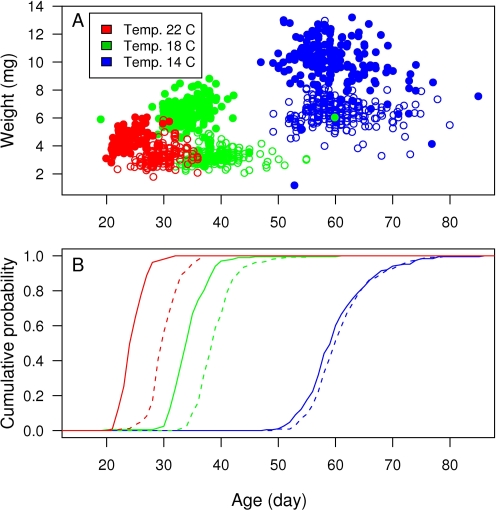
Timing of metamorphoses of common frog (*Rana temporaria*) reared in a common garden experiment. Individuals are exposed to three temperature and two food level (*ad libitum* or restricted) treatments. In both panels, growth temperatures are indicated with colours (see colour legend), and restricted food is indicated with open circles/dashed line, and *ad libitum* feeding with filled bullets/solid line. Individual observations of ages and weights at metamorphosis are shown in panel A. Cumulative probabilities for the timing of metamorphoses calculated from the raw data are shown in panel B.

In the experiment, individually reared tadpoles from one study population located in central Sweden (Umeå, 36°49′ N, 20°14′ E) were exposed to three temperature treatments (warm: 22°; medium: 18°; cold: 14°) and two food level treatments (restricted and *ad libitum*), and their weight and age at metamorphosis were recorded ([27]; Fig. 1). As the individuals were obtained from artificial crosses, their genetic relationships were exactly known, allowing the estimation of additive genetic variance in transition probabilities (see below). Details of the experiment and rearing conditions of the animals can be found from [27]–[28].

In the analyses, we focused on investigating how the timing of metamorphosis depends on temperature and food level. These variables were treated as factors. We did not include body size into the analyses as its role in determining the timing of metamorphosis was clearly insignificant: size at metamorphosis varies widely among individuals exposed to different treatments (Fig. 1), suggesting that the treatments affect the process of metamorphosis rather directly than through body size. In addition, as any systematic patterns in growth between the treatments is induced by the treatments themselves, patterns in growth are correlated with the treatments, and having body size as well as treatments as explanatory variables could bias the analyses.

To compare model performance with different data types, both survival models were fitted with time was treated both as continuous and censored with a 10 day interval (eqn 3 and 4). In the parametric survival model (eqn 3) survival time was considered as a logistically distributed variable using an identity link function [16]. We also tried Gaussian, Weibull and exponential distributions, but these provided fits very similar to that of logistic distribution so that we restricted to present results only for the logistic distribution. In the Cox regression (eqn 4), the restricted food level and cold temperature were considered as baseline conditions. To investigate how frailties would change the picture, we also added normally distributed frailties to the parametric survival model (eqn 3) as a sire effect. The additive genetic variance in the timing of metamorphoses could then be estimated as four times the sire variance [25].

**Figure 2:**
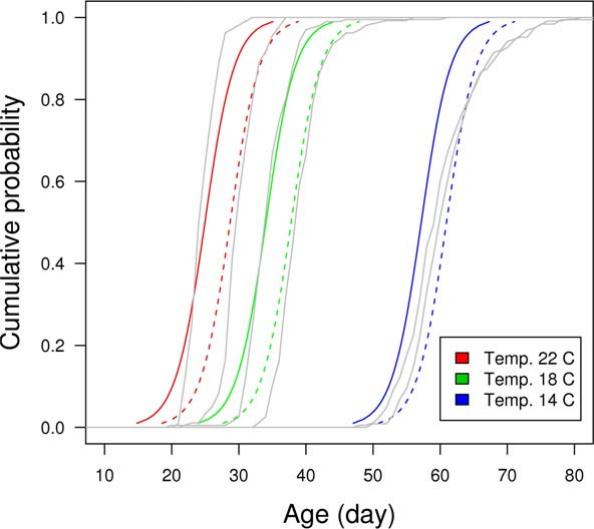
Cumulative probabilities for the timing of metamorphoses predicted by the parametric survival model (eqn. 3). Different growth temperatures are indicated with different colours, and different food level treatments with different line types (solid line = *ad libitum*, dashed line = restricted food). Gray lines beneath the estimated cumulative probabilities are the observed cumulative distributions for the timing of metamorphosis.

The parametric survival model (eqn 3) turned out being flexible in describing the effects of environmental treatments on the timing of metamorphoses. When time was considered as continuous, the model predicted that 50% of the frogs would metamorphose by the age of ca. 61 days when being exposed to cold temperature and restricted food. *Ad libitum* feeding conditions were predicted to shift the location of the median to four days earlier, whereas medium and warm temperatures caused shifts of the location 23 or 32 days earlier, respectively (Table 1). These predictions matched rather well with the timing of metamorphoses observed in each treatment group, with largest deviations between model predictions and observed patters being mainly in the beginning and in the end of each metamorphosis pattern (Fig. 2). Treating time as interval censored did not change the model parameter estimates much, although the standard errors increased (Table 1), suggesting that the parametric survival model (eqn 3) is robust with respect to the time resolution of observations of the transition events.

**Table 1:** Model parameters and their standard errors (se) estimated with the parametric survival model (eqn 3) as applied to common frog data.

	Continuous time			Time intervals (10 day)		
	parameter	se	p-value**	parameter	se	p-value**
α*	60.94	0.227	<0.01	60.04	0.258	<0.01
Food *ad libitum*	−3.76	0.223	<0.01	−2.39	0.299	<0.01
Temp. 18 C	−23.14	0.264	<0.01	−23.07	0.333	<0.01
Temp. 22 C	−32.23	0.290	<0.01	−31.94	0.368	<0.01
σ*	2.2	0.025	<0.01	2.21	0.034	<0.01

* Symbols α and σ are the intercept and scale parameters in eqn 3.

** P-values are derived from z-tests.

Similarly, the Cox regression model (eqn 3) also captured the environmental variation in the timing of metamorphoses. Parameter estimates for the continuous time model (Table 2) clearly show that *ad libitum* food and medium and warm temperature levels increase the probability of metamorphosis relative to that in restricted food level and cold temperature. For example, if being exposed to medium temperature and *ad libitum* feeding conditions, the decrease in the probability of not being metamorphosed by time *t* in contrast to the baseline probability (restricted food level and cold temperature) could be described by −log (P(*T*>*t*)) = P_0_ (*t*)×2.18×38.9 (Table 2). In all, Cox regression model explained the data well, by incorporating 82.3% of variation in the timing of metamorphoses (standard R^2^). When *T* was interval censored, the explanatory power of Cox regression was nearly as good with R^2^ being 74.4%.

**Table 2:** Model parameters and their standard errors (se) estimated for the semi-parametric Cox regression survival model (eqn 4) as applied to common frog data.

	Continuous time			Time intervals (10 day)		
	exp(parameter)	se	p-value*	exp(parameter)	se	p-value*
Food *ad libitum*	2.18	0.066	<0.01	1.68	0.063	<0.01
Temp. 18 C	38.90	0.116	<0.01	23.88	0.103	<0.01
Temp. 22 C	641.25	0.156	<0.01	129.23	0.131	<0.01

* P-values are derived from z-tests.

In the parametric survival model the estimated sire variance for continuous time data was 2.63, giving an estimated additive genetic variance (V_A_) of 10.5 over all the treatments. This is roughly the same order of magnitude as the food effect: a difference of one standard deviation in the genetic quality (i.e. the breeding value) of an individual is 3.24, only slightly less in magnitude than the food effect (−3.76). The sire effect for the analysis with the 10 day interval censored time data was 2.79, so the censoring has little effect on the point estimate.

## Discussion

The models for probabilistic description of ontogenetic life-history transitions presented in this study are convenient in the sense that they can be readily fitted to an observed pattern of transition events without tight requirement of information of ontogenetic trajectories or temporal changes in covariates. In that sense these models are of empirical type, aiming at describing the transition pattern and how it is shifted by the covariates, rather than pursuing (semi-)mechanistic description of causality between covariates and transition rates as is done in the more sophisticated model by van Dooren et al. [14]. These features of our models allow for straightforward modelling of such data that might in practice be typically available from wild, as well as for predicting transition patterns relatively easily.

Simplicity of the models obviously does not come without trade-offs: the lack of causal description of covariate effects on transitions rates (i.e. the hazard) means that the models also do not provide very detailed information about the mechanisms underlying the individual transitions, but rather, describe average effects of covariates on the entire transition patterns. Therefore, the models provide a tool to approximate patterns of transitions in a population and how those change if average environment changes, but for a detailed description and prediction of unique ontogenic trajectories and life-history strategies, the models capacity may be limited. Also, our models can be oversimplified if covariates vary strongly in time in a manner that cannot be encompassed by any choice of a parametric survival time distribution (i.e. approximated by the form of hazard underlying the chosen survival time distribution), or if the covariate trajectories are very unique for each individual, so that the stochasticity in the transition pattern cannot be encompassed by the random part of the model. For these cases the transition rate based model developed by van Dooren et al. [14] provides a better solution, but also requires both more detailed and often individual specific data, and much modelling effort to construct the analytical hazard function. Consequently, we find the models suggested in this study and the model developed by van Dooren et al. [14] to be complementary rather than competing. First, our models can be utilized as a first step to model ontogenetic transitions and, if their fit is not considered sufficient, a more detailed model for the underlying hazard [14], [29] can be constructed based on the information the simple models provide about the shape of the pattern and how it is in average affected by covariates. Secondary, sometimes data is not sufficient to provide detailed information about a process, so the simplified models described here provide a way to utilize the information that is available. If so, these models may also help the assessment of how detailed data has to be to understand and predict the full process, and to point out the most relevant mechanisms on which empirical effort can then be focused.

In the comparison with empirical data on the timing of metamorphoses of common frog, the parametric survival model was able to describe the pattern of metamorphoses rather well (Fig. 2), and Cox's regression model showed its capability in detecting relative roles of temperature and food in the timing of metamorphoses (Table 2). Of course, as the experiments were carried out in controlled laboratory conditions, the data may include less uncontrolled variation that would be expected in wild. Furthermore, covariates (temperature and food) were kept constant over the developmental period, which would hardly be a natural situation. Therefore, even though performing well in this study, it would be informative to investigate the model performance with datasets collected from the wild. Despite its limitations the data used in this study demonstrates a situation in which the simple survival models developed here might be preferred over the traditional discrete time PRNs [7] and the more sophisticated model by van Dooren et al. [14]. In the case of discrete time PRNs, time should have been split into intervals wide enough to encompass that many metamorphoses that the proportion of metamorphosed individuals would reliably reflect the underlying probability of metamorphoses. Predictions about the metamorphoses could have been possible only within the same fixed intervals and they would simply reflect the proportion of metamorphoses seen in the data within the same interval, whereas the parametric survival regression provides a continuous time model for the underlying metamorphosis process (Fig. 2) and allows one to derive predictions for any freely chosen interval. In contrast, the model of van Dooren et al. [14] may be too complicated way to start to analyse the data: in the absence of strong prior knowledge of the ways in which covariates affect the metamorphoses rate, one should integrate the survivor function for several choices of hazard functions (eqn 2) to compare which of them would provide best fit to the data. Therefore, the simple survival models appear to provide a prompt but still fairly realistic way to illustrate, analyze and predict the observed patterns of metamorphoses in continuous time.

PRNs for the age and size at maturation have rapidly become the tool-of-trade in studies of fisheries-induced evolution [9], [30]–[31]. To date, PRN analyses have been performed for more than a dozen fish stocks (reviewed in [9]). These studies have looked at long-term shifts in maturation in fish stocks with naturally periodic maturation process [7]–[8], so that the logistic regression based discrete time PRNs have been adequate in describing maturation. Yet continuous time models have been developed for the transition processes before [14] these have not become similarly established modelling tools. The models presented in this study make the concept of probabilistic modelling of ontogenetic life-history transitions in continuous time more easily accessible, by offering easy-to-use tools for analysing transition data typically available from wild with little prior knowledge of mechanisms underlying transitions. Likewise, by allowing easy incorporation of random effects into the models, the survival analysis based models can be applied to estimate genetic variability in transition probabilities, given that the data contains the needed pedigree information. Such data could be obtained from breeding (e.g. aquaculture) experiments, or from data collected with aid of genetic markers (e.g. [32]). These features should make the survival based models useful e.g. in the studies investigating and predicting patterns of metamorphosis in insects and amphibians. More generally, they should aid the concept of probabilistic reaction norm becoming as general and applicable tool in the studies of life-history variation as the deterministic reaction norms are today.
